# Big Sound and Extreme Fungi—Xerophilic, Halotolerant Aspergilli and Penicillia with Low Optimal Temperature as Invaders of Historic Pipe Organs

**DOI:** 10.3390/life8020022

**Published:** 2018-06-14

**Authors:** Katja Sterflinger, Christian Voitl, Ksenija Lopandic, Guadalupe Piñar, Hakim Tafer

**Affiliations:** VIBT-Extremophile Center, University of Natural Resources and Life Sciences Vienna, Muthgasse 18, A-1190 Vienna, Austria; christian.voitl@boku.ac.at (C.V.); Ksenija.lopandic@boku.ac.at (K.L.); guadalupe.pinar@boku.ac.at (G.P.); hakim.tafer@boku.ac.at (H.T.)

**Keywords:** pipe organs, xerophilic fungi, biodeterioration

## Abstract

Recent investigations have shown that xerophilic fungi may pose a biodeterioration risk by threatening objects of cultural heritage including many types of materials, including wood, paint layers, organic glues or leather and even metal. Historic—and also new built—pipe organs combine all those materials. In this study, halotolerant aspergilli and penicillia with low optimal temperatures were shown to be the most frequent invaders of pipe organs. The fungi form white mycelia on the organic components of the organs with a clear preference for the bolus paint of the wooden pipes, the leather-made hinges of the stop actions and all parts fixed by organic glue. Physiological tests showed that the strains isolated from the instruments all show a halotolerant behavior, although none was halophilic. The optimum growth temperature is below 20 °C, thus the fungi are perfectly adapted to the cool and relatively dry conditions in the churches and organs respectively. The *de-novo* genome sequences analyses of the strains are currently ongoing and will reveal the genomic basis for the halotolerant behavior of the fungi.

## 1. Introduction

**Figure 1:**
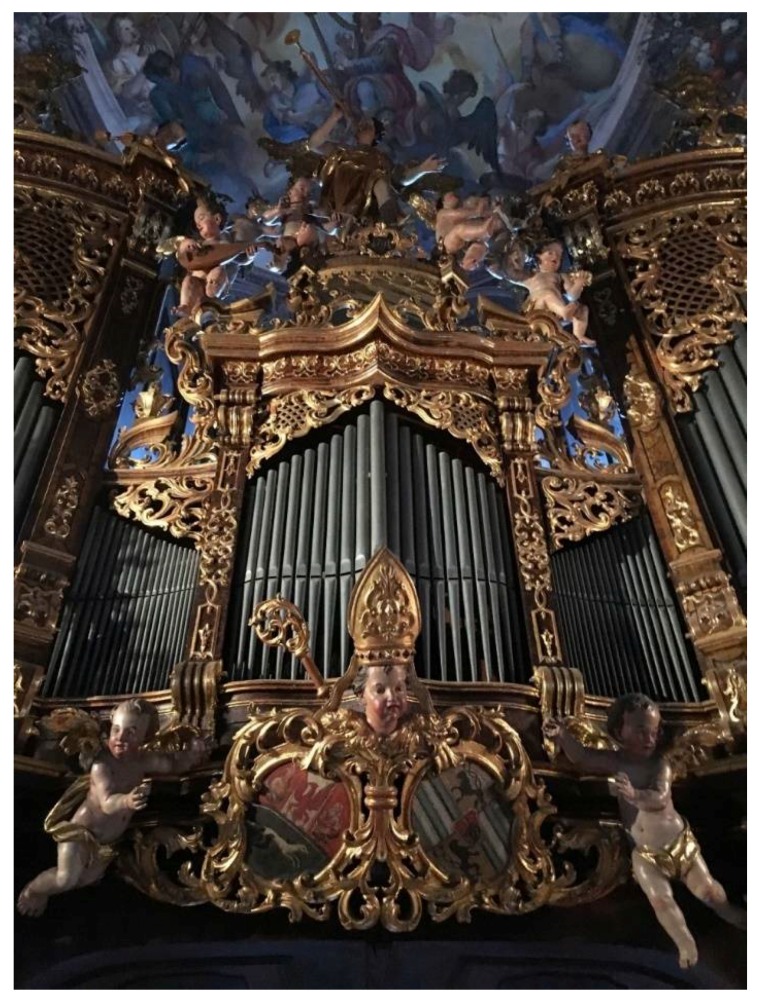
Baroque pipe organ with decorations (Styria, Austria).

Pipe organs are certainly one of the oldest and most complex musical instruments; several hundreds of wooden and metal pipes are selectively driven by a complex mechanical machinery directing the wind by means of different windchests, stops, trackers, pallets, valves and wires. Pipe organs are an important part of sacral music and sacral liturgies. Today, organs still have an important function in churches and many historical organs are still in use. Modern and impressively large instruments are located in the Philharmonie of Paris (the organ was built in 2016), in the Lotte concert hall in Seoul (KR) (built in 2016) and in the Holy Sepulchre Church in Jerusalem (built in 2014). Pipe organs are examples of living cultural heritage and the basic techniques and mechanics of an organ remain the same in the new built organs [1,2]. The major components of an organ are made of metal, wood, paint layers, leather and glue. The instrument itself is housed by the organ’s case, which is usually made of wood and often equipped with splendorous ornamentation and paintings (Figure 1). The construction of an organ is a real art and high quality craft technique. Organs have always been constructed with respect to the architecture and acoustics of the church or room for which it was planned [3]. This also includes consideration of the indoor climate of the church, the air current, the sun exposure of the organs through windows and the location inside the church where the air steam for the organ is collected. In old times, when the wind in the organs was produced by mere human action on bellows, the air was taken next to the organs. Later, when human power was replaced by electrical air blowers, those blowers have been moved to adjoining rooms, e.g., the sacristy, because of the noise of the motor. The air for the organ then was taken from these adjoining rooms with different possible temperatures and humidity levels as compared to the organs’ case (personal communication, master organ builder Siegfried Adlberger). The organ, as a complex of pipes and wind-mechanics, thus can be regarded as a complex entity with different climatic niches. 

In principle, organs in churches do not provide the best environment for the growth of micro-organisms: the temperature in a church is usually low, often not exceeding 20 °C even during the hot summer months and only slightly above 0 °C during the winter. Moreover, the air current inside of an organ usually causes a low water availability and organic nutrients are limited to glued connections within the trackers and a small number of painted wooden pipes. However, within the last 15 years reports on mold growing inside of organs have been increasing rapidly [4], (master organ builder Siegfried Adlberger, personal communication; Schimmelpilzsanierung im Orgelbau, Bund Deutscher Orgelbaumeister e.V.). It seems that mold in organs is an increasing problem in both historical and new instruments. Thus, it was the aim of this study to investigate a number of pipe organs in churches with special focus on their fungal contamination, biodeterioration phenomena caused by fungi, to characterize the ecology of the fungi and to evaluate if the fungal contamination poses a health risk for humans by the release of spores. To address this aim, six organs located in different churches of different regions of Austria were sampled, the fungi were isolated and identified based on morphology and DNA sequencing and the predominant species were tested with respect to their xerotolerance.

## 2. Materials and Methods

### 2.1. Sampling and Objects

Samples were taken from the following pipe organs located in different churches in rural areas in Austria and one from the city of Vienna:

- parish church St. Nikolaus, Bad Ischl (Upper Austria)
- parish church Waldzell (Upper Austria)
- parish church Eggelsberg (Upper Austria)
- parish church Schwanenstadt (Upper Austria)
- parish church Altheim/St. Laurenz (Upper Austria)
- parish church Heiligenstadt, Vienna (Vienna)

The organs of the parish church Eggelsberg and the church in Waldzell are recent instruments built in 2001 and 1998 respectively. The other instruments are historical instruments from the 19th and beginning of the 20th century. 

**Figure 2:**
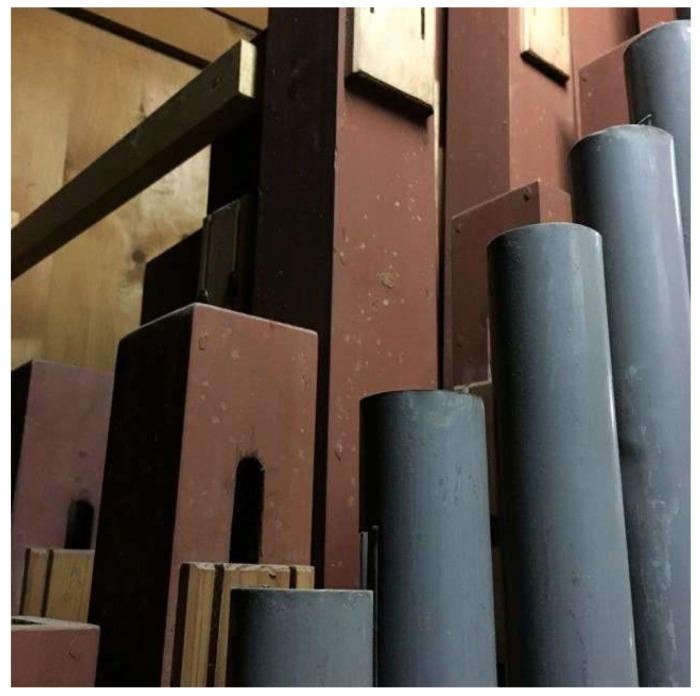
Light fungal colonies on Bolus painted wood pipe.

**Figure 3:**
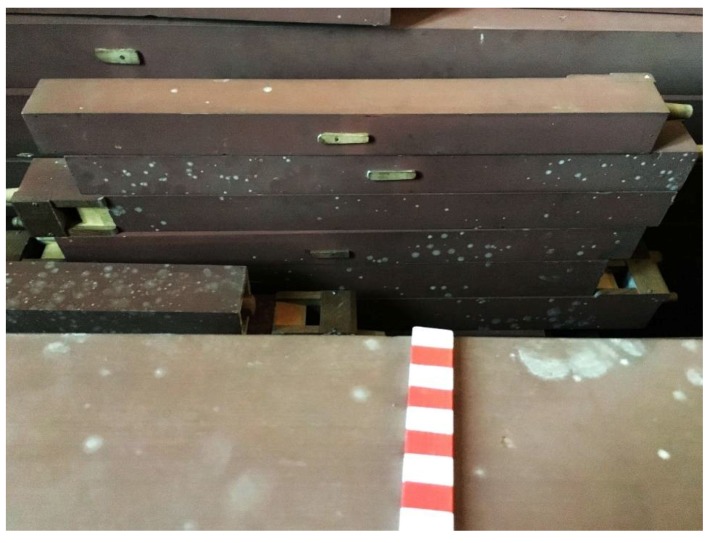
Heavy fungal deterioration of wood pipes.

**Figure 4:**
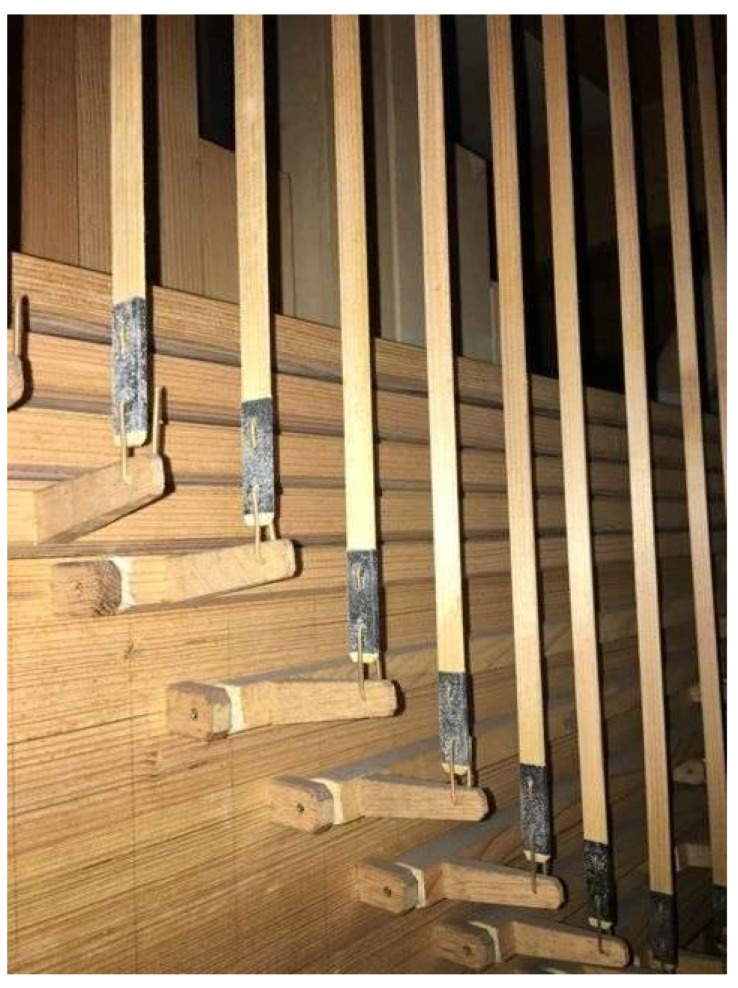
Fungal growth on hinges of stop actions.

**Figure 5:**
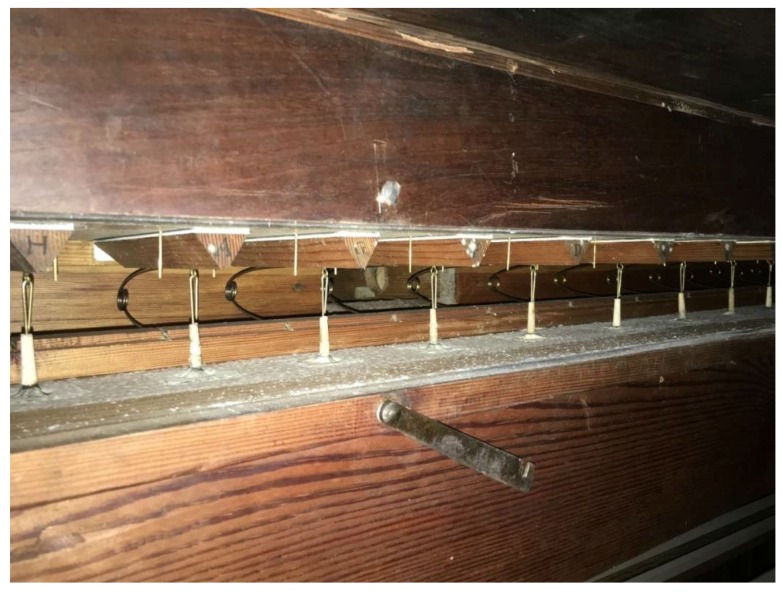
Fungal contamination on leather sealings (“pulpetes”) within the windchest.

**Figure 6:**
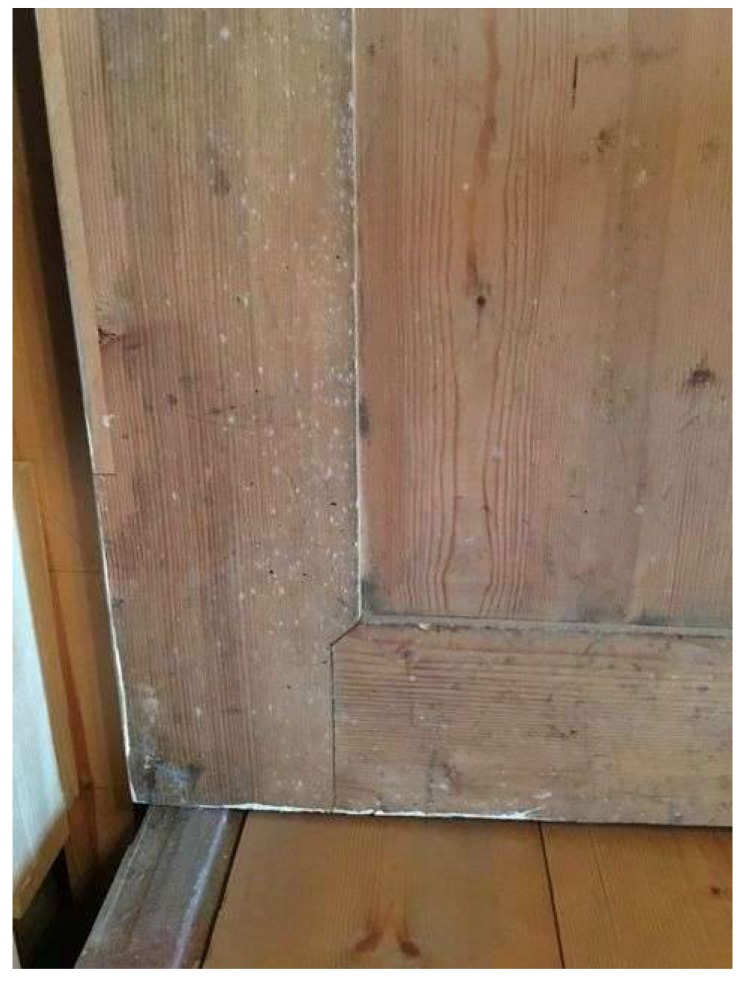
Fungal growth on the inner part of the organs casing.

Samples were collected from different representative surfaces inside of the organ’s case, on its outside, on the surface of the pipes, on the tracks and inside of the windchests where fungal contamination was visible with the naked eye (Figure 2, Figure 3, Figure 4, Figure 5 and Figure 6). The sampling was carried out non-invasive using commercial contact plates as follows: two plates of 2% Malt Extract Agar (MEA) and two Dichlorane Glycerol + chloramphenicol (DG18) agar (55 mm, Heipha, Eppelheim, Germany) per sampling point. The plates were opened and the agar surface was evenly pressed onto the dry surface of the wood for a maximum of two seconds. Afterwards the plates were closed and brought to the laboratory for incubation. In addition surface samples were taken with sterile swabs for enrichment cultures for the isolation of fungi tolerating low water activity (a_w_): Salt Agar (SA), containing 0.1% malt extract, 0.67% Nitrogen base, 5% glucose, 2% agar, added to 10% (*w*/*v*) NaCl. These plates and the obtained contact plates were incubated at room temperature (20–22 °C) for between 9 and 30 days, depending on the growth of fungi. Grown colonies were transferred to fresh plates containing MEA and SA media to obtain further pure isolates. The pure isolates from MEA and DG18 were then identified based on their macro- and micromorphology. The morphological identification of fungi was carried out according to Pitt [5], Klich [6], Samson et al. [7], de Hoog et al. [8], Bensch et al. [9,10], Samson et al. [11], Sklénar et al. [12]. Pure isolates grown on SA were incubated at room temperature (20–22 °C) until the mycelium was sufficiently grown for DNA-extraction (20–48 days).

To link the NaCl concentration with aw and sucrose, a table published by the Food Safety Bulletin was used [13]. Temperature profiles for each salt concentrations were 5 °C, 10 °C, 15 °C, 20 °C, 28 °C and 37 °C.

### 2.2. DNA Extraction and Sequencing

The DNA extraction of pure fungal strains was performed according to the CATB based protocol by Sert and Sterflinger [14]. All PCR reactions were performed with the 2× PCR Master Mix from Promega (Vienna, Austria), 50 units/mL of TaqDNA Polymerase supplied in an appropriate reaction buffer (pH 8.5), 400 µM dATP, 400 µM dGTP, 400 µM dCTP, 400 µM dTTP, 3 mM MgCl, which was diluted to 1×, and 12.5 pmol/µL of each primer (stock: 50 pmol/µL, VBC-Biotech, Vienna Austria) were added. In a total volume of 25 µL, 400 µg/mL BSA (stock: 20 mg/mL; Roche, Diagnostics GmbH, Mannheim, Germany) and 2.5 µL DNA template were added. All PCR reactions were executed in a BioRad C1000 Thermal Cycler (Hercules, CA, USA). The entire reaction batches were run with 4 µL loading dye solution (Fermentas, Waltham, MA, USA) in a 2% (*w*/*v*) agarose gel for ~130–160 min at 70 V, stained in an ethidium bromide solution (1 mg/mL; stock 10 mg/mL) for 30 min and visualized by a UVP documentation system (BioRad Transilluminator, Universal Hood; Mitsubishi P93D-printer, Cypress, CA, USA). The GeneRulerTM 100 bp DNA ladder (Fermentas) was used as a size marker. For sequencing of fungal isolates, the primer pair ITS1 and NL4 were used. The thermocycling program used was as follows: 1 min denaturation at 98 °C, followed by 35 cycles consisting of 30 s denaturation at 98 °C, 30 s primer annealing at 60 °C and 105 s primer extension at 72 °C, followed by a final extension step of 2 min at 72 °C. 

All PCR products obtained were purified using the QIAquick PCR Purification Kit (Qiagen, Hilden, Germany) and analyzed by electrophoresis in 2% (*w*/*v*) agarose gels. PCR products were externally sequenced by Sanger sequencing with a fleet of 16 ABI 3730xl (GATC Biotech, Konstanz, Germany). Comparative sequence analyses were performed by comparing pairwise insert sequences with those available in the online databases provided by the National Center for Biotechnology Information using the BLAST search program [15]. The sequences retrieved from all isolated strains have been deposited in the NCBI nucleotide database under the accession numbers listed in the results.

#### Whole Genome Sequences and Bioinformatics Analysis

Genomic DNA was isolated from the strains MA6036, MA 6037, MA 6038, MA 6039, MA 6040 and MA6041 on SA using a cetyltrimethylammonium bromide (CTAB)-based protocol [14]. Genome sequencing was carried out using the Ion Torrent technology (Ion PGM Hi-Q View kit; Life Technologies, Inc., Carlsbad, CA, USA), according to instructions of the manufacturers. The genomes were assembled with Newbler 2.9. RRNA sequences used for the taxonomic classification were found in the genome with cmscan [16] and the 28S, 5.8S and 18S Rfam model [Rfam13.0: shiftingtoagenome–centricresourcefornon–codingRNAfamilies], while the gene sequences for Tef1 and ß-tubuline were identified with BLAST [https://www.ncbi.nlm.nih.gov/pubmed/20003500?dopt=Citation]. 

The Whole Genome Shotgun projects have been deposited at DDBJ/ENA/GenBank under the accession PQME00000000 (MA6038), PQMF00000000 (MA6039), and QAGG00000000 (MA6036), QAGH00000000 (MA 6037), QAGI000000000 (MA6040), QAGJ00000000 (MA6041).

### 2.3. Cardinal Values of Fungal Isolates from Salt Agar

Strains were incubated on SA with the following concentrations of NaCl: 0%, 5%, 10%, 15%, 20%, 25% and 30%; each condition was gain combined with different temperatures: 5 °C, 10 °C, 15 °C, room temperature, 28 °C and 37 °C. The colony diameter was measured after 7, 14, 21 and 42 days. Each experiment was carried out in triplicate.

### 2.4. Viable Spore Count in the Indoor Air

In order to analyze if the fungal contamination of the pipe organs influenced the indoor air quality, the number of viable and cultivable fungal spores and mycelial fragments in the air was measured according to the mold-guideline be the German and Austrian Ministries for Health and Environment [17]. Measurements were carried out using the EcoMAS 100 (Merck, Kenilworth, NJ, USA) and sampling of 100l air volume. Measurements were carried out close to the organ, especially at the place of the organist and were distributed over the nave of the church. The number of measurements thus depended on the size of the churches ranging from 10 to 15 measurements distributed equally over the nave and the gallery of the churches. Media used were MEA, DG18 and SA agar with 10% NaCl. Plates were incubated 7 (MEA, DG18) up to 48 days (SA) at room temperature and colonies were counted.

## 3. Results

The major components of the organs are the wooden and metal pipes, the wind load, the mechanical note- and stop actions and the swell organ. These components are enclosed in the organ’s case, which has two major functions: (1) sheltering of the organ’s sensitive components and (2) to hold together and to amplify the sound of the instrument. The organ’s case provides a special microclimate for the growth of fungi that is again influenced by the climate of the church in which the organ is located.

### 3.1. Biodeterioration Phenomena

Although the instruments and churches are quite different and the age of the pipe organs varies from 15 to 130 years, the deterioration phenomena displayed were very similar: Fungi were growing preferentially on the wooden pipes that have a polychrome surface made of Bolus (Figure 2). The fungi were forming light white or ochre colonies on the paint layers. Only in case of one church, namely Heiligenstadt, greyish-green colonies were additionally found on the bolus pipes (Figure 3). The next location where fungi were preferentially growing are the leather connectives and hinges within the trackers (Figure 4). In addition, the leather sealings named “pulpete” on the bottom of the windchests showed fungal growth (Figure 5). Out of all the pipe organs, only white and ochre mycelia were observed on these materials. Significantly less fungal growth was observed on the wooden pipes without paint, and no growth was found on the metal pipes. In some rare cases, single fungal colonies were found on the inner surfaces of the casing (Figure 6). Also here, only white punctual colonies were observed.

### 3.2. Fungal Isolates and Their Identification

The results of the cultivation experiments and the identification of the fungi are shown in Table 1. A total of 39 fungal strains could be isolated from the seven pipe organs. Isolates growing merely an MEA and DG 18 were identified morphologically using the identification keys. Isolates able to grow on 10% NaCl medium were additionally identified based on partial 18S rDNA and ITS sequencing data (Table 2). The fungi found in the pipe organs can be divided into two ecological groups: The first group comprises fungi found ubiquitous in the environment, as common aspergilli and penicillia and species of *Cladosporium*, *Acremonium*, *Alternaria* and *Fusarium*. These fungi need water activities higher than 0.85 a_w_ and thus are not xerophilic. The second group are those able to grow on SA with 10% NaCl. The eight different isolates were morphologically grouped into the genera *Penicillium* and *Aspergillus* (Table 2). Sanger sequencing of the ITS regions with a subsequent homology search using Standard Nucleotide Blast did not allow the assignment to any known species. In the following step, full genome sequences were analyzed targeting the following regions: Tef1, ß-tubuline, 18S (using the databases of NCBI and SILVA) and the ITS regions (Table 3). The results gained from this analysis were not homogeneous. It can be carefully concluded that strain MA 6040 is close to *P. rubens*, strain MA 6041 shows high similarity to *Aspergillus vitricola* and strains MA 6038 and 6039, albeit isolated from different churches and organs, show a high similarity with each other and with *A. glabripes*. Strains MA 6036 and MA 6037 could not be assigned to a known species and must be probably described as new species of the genera *Penicillium* and *Aspergillus* respectively. The different results obtained from the taxonomic assignment between ITS sequences data, derived from Sanger sequencing and from the WGS by high throughput sequencing, can possibly be explained by the fact that about 20% of the fungal ITS sequences are incorrectly identified to the species rank [18], that taxonomical assignment at the species level is dependent on the selection of the DNA markers [19], and that sequencing errors might lead to incorrect ITS/marker sequences. 

### 3.3. Cardinal Values of Fungi Isolated on NaCl Medium

**Figure 7:**
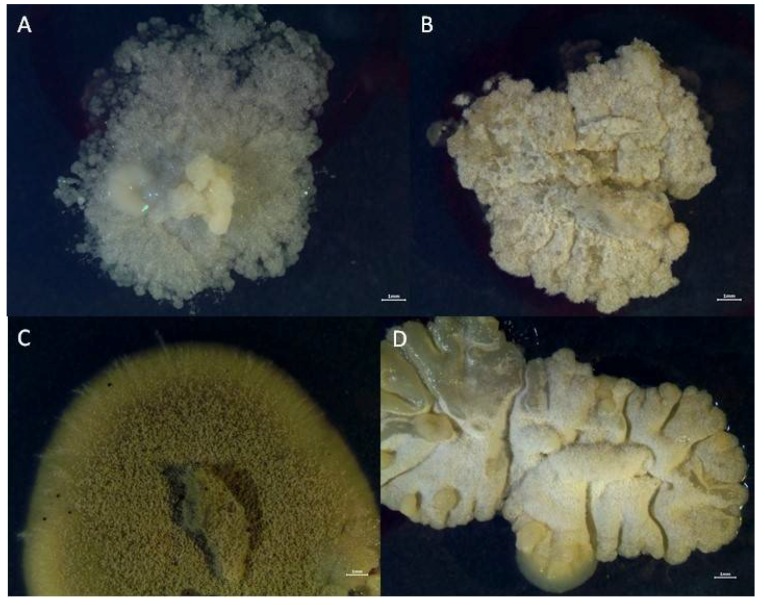
Colony morphology of the four dominant stains grown on a maximally tolerated salt concentration: (**A**) MA 6036, *Penicillium* sp., 20% NaCl; (**B**) MA 6037, *Aspergillus* sp., 25% NaCl; (**C**) MA 6039, *Aspergillus* sp., 15% NaCl; (**D**) MA 6040, *Penicillium rubens*, 25% NaCl.

The cardinal growth values of the isolates were tested at 5 °C, 10 °C, 15 °C, 20 °C, 28 °C and 37 °C and in combination with NaCl concentrations from 0 to 30% in steps of 5%. The summary of results is shown in Table 4. All four species were shown to have their optimal growth at a temperature of 15 °C to maximum of 20 °C. None of the fungi tested was halophilic but all tolerated NaCl concentrations of at least 15%. Strain MA 6039 (possibly *A. glabripes*) tolerated 15% and *Penicillium rubens* tolerated 25% of NaCl in the growth medium. Thus, the fungi in the organs can be classified as xerophilic halotolerants with a favor for temperatures lower than room temperature. All species tested formed white or ochre colonies without any typical sporulation, or green, grey-green or bluish green pigmentation when cultivated on SA (Figure 7).

### 3.4. Indoor Air Measurements

The amount of spores in the indoor air of the churches is shown in Table 5, with maximum and minimum values of 10 to 15 measurements equally distributed through the nave and close to the organ. The amount of fungal spores in the indoor air is in the size range of the outdoor reference measurement in all churches. Only in Bad Ischl was the number of spores raised in close vicinity to the organist’s place. Only in this church, halotolerant isolates were found on the plates. In the nave of this church, as in the other five churches, the indoor air reflected the amount and the diversity of the outdoor air, which was measured as a reference and showed no signs of being influenced by the fungal contaminations of the organs.

## 4. Discussion

Fungal growth on organs and other pieces of cultural heritage not only cause serious aesthetical spoiling due to colony formation and fungal pigments. Fungal growth also degrades materials and thus affects objects substantially [20]. The enzymatic degradation of organic binders causes reduction of paintings; fungi penetrate cracks and migrate underneath paint layers thus causing detachment. In paper and wood conservation, fungi are a particularly major problem due to their ability to excrete cellulases. The fungal degradation of organic glues, as e.g., rabbit glue, can even lead to the collapsing of whole organ pipes [4]. In pipe organs, fungi pose enormous difficulties for restorers and organ builders. Many parts of the organs—as the interior of the pipes or the cancelles within the windchest—are extremely difficult to reach and to clean. 

The results of this study have shown that the interior of pipe organs provides a suitable habitat for fungi that are adapted to low water availability. Although a number of ubiquitous hyphomycetes with demand for water availabilities above 0.85 were isolated on MEA and DG 18, the picture in the organs suggest that these fungi did not really thrive in the instruments. The white mycelia found on the organs surfaces are typical for halotolerant fungi when growing under conditions of low a_w_ but not for species of *Cladosporium* or *Alternaria*. The latter were rather isolated on the plates as transients, because of airborne spores in dust layers. The climate in the churches and organs seems to be selective for fungi adapted to low water availability and the fungi, in turn, can be regarded as climate indicators in this case. Generally, growth of fungi in indoor environments is largely determined by the indoor climate, the amount of available nutrients—from the atmosphere and from the materials themselves—and also by the cleaning intervals [20]. Depending on the climate in a church, a museum or a storage room, fungal diversity is restricted to few xerophilic and xerotolerant species. Only in environments where the relative humidity is raised to more than 85% for a period of several days, high fungal diversity is able to establish itself. The climate ranges allowing fungal spores to germinate and that restrict the growth of the fungal mycelium are shown in the isopleth systems by Sedlbauer & Krus [21]. The authors also show that hygroscopic materials can support the growth of fungi at low relative humidity and that the water demand depends on the biodegradability of the substrate. The objects influence the development of the fungal community by their chemical composition and biodegradability for species with different exoenzymes. The pipe organs’ microflora investigated in this study was clearly dominated by fungi with a high tolerance towards low water availability. The preference for the Bolus paint and for the leather hinges and glue is clear since these substrates provide a carbon source for the fungi. Bolus is a red ochre or grey mineral that is mixed with organic binders, like animal glue or egg white. Bolus can be used as a basis for leaf gold surfaces or a polychrome surface for wooden pipes where it has an influence on the acoustic color/timbre of the pipe (personal communication maser organ builder S. Adlberger). Animal glue—also used for the leather connectives and hinges—and egg-white have the ability to be hygroscopic and thus provide not only nutrients to the fungi but also higher water availability, which is the case in the other parts of the organ. 

Although a final assignment of the isolated strains to a species was not possible and at least two strains might represent new species, it can be concluded that xerophilic aspergilli and penicillia dominate the actively growing myco-flora in the pipe organs. Both genera have been described to comprise a number of halotolerant and halophilic xerophiles [22,23,24]. *Aspergillus vitricola* was first isolated from glass surfaces [23] and occurs frequently in house dust. On archive material, the fungus was observed to form thin white mycelial colonies resembling those found in the pipe organs [25]. *Aspergillus glabripes* again also occurs on books and archive material as well as in house dust [26]. Also *A. glabripes* forms white mycelia on the materials. Both fungi are taxonomically close and belong to the *Aspergillus* section *Restricti*, which comprises xerophilic species that are able to grow on substrates with low water availability and in extreme habitats [22]. Some species of the section *Restricti* were isolated from hypersaline waters. However, *A. vitricola* was not found in this environment which can be explained by the fact that it is not a real halophilic but halotolerant species as show in this study. The third halotolerant fungus found in the pipe organs was the species *Penicillium chrysogenum*/*P. rubens* respectively. The two species are phylogenetically very close and cannot be distinguished based on ITS data. Only recently, the first producer of Penicillin was re-identified as *P. rubens* rather than *P. chrysogenum* [27]. *Penicillium rubens* is commonly found in indoor dust and air. The critical a_w_ for *P. rubens* under ideal circumstances, i.e., on MEA, has been found to be 0.79 [28]. *Penicillium chrysogenum* in contrast seems to be very tolerant against low a_w_ values and has been even reported from hypersaline waters in the Adriatic and Eilat salterns [23]. 

A fungus with similar ecology to those isolated from the pipe organs, which has recently been reported as a “biological invasion” on library materials, is *Eurotium halophilicum*. *Eurotium halophilicum* is an extremely xerophilic species, also regarded as pioneer species on archive materials [25]. *Eurotium* species are perhaps the epitome of xerophilic fungi, being capable of rapid growth over wide temperature and aw ranges (minimum ~ 0.70–0.72 a_w_), and having a cosmopolitan distribution [28]. The in-situ morphology of the fungus on objects is quite similar to the morphology of the species found in this study. However, the fungus was not isolated from the organs, although it is frequently found on archive material and in indoor environments in Austria too (Sterflinger & Voitl, unpublished data). One possible explanation could be that in the churches and organs, fungi with low optimal temperatures have an advantage as compared to *Eurotium halophilicum*, which has its optimum temperature for germination at 30 °C [29]. The optimal growth temperature for the fungi found in the organs was 15 °C for *Aspergillus glabripes* and *Penicillium chrysogenum*/*rubens* and 20 °C for *A. vitricola* respectively.

As mentioned above the biocolonization phenomena in all pipe organs analyzed (and in many other organs in Austria, Sterflinger unpublished data) are very similar: White mycelia are especially covering those surfaces where organic nutrients, as bolus paint, leather or animal glue, are available. In few cases, plain wooden surfaces are also inhabited by the fungi, again with white punctual or more spreading colonies. The general situation is that the humidity inside of the organs’ cases is slightly too high. In contrast, a significantly high RH with values more that 70% RH would favor the growth of more fungal species with need for higher a_w_ values. In Slovakian churches, a much higher diversity of fungi was reported to inhabit the instruments [4]. In all churches observed, small building changes led to a change of the climate, especially ventilation: E.g., installation of new doors, closing of the holy ghost openings in the ceilings or installation of sheltering glass for the church windows and the installation and use of heating systems. Thus, the most important measure to prevent fungal growth inside of the organs is a sound knowledge about the recent history of building measures inside the churches and of building physics in general. Climate monitoring has to be carried out in the churches and inside of the organs.

The cleaning measurements recommended for the pipe organs are as follows: (1) mechanical cleaning by hovering with high-efficiency-particle absorbers can remove superficial mycelia and spores if present; (2) mechanical re-movement of attached mycelia using sponges or adhesive rubbers regularly used by restorers. A treatment with 70% isopropanol is recommended with a minimum of two minutes of reaction time. This however, can only be done on surfaces not sensitive for dissolution by alcohol. On surfaces sensitive to an alcohol disinfection a solution of 1% benzalconiumchloride with <0.1% isothiazolinone in water can be applied. Studies on the sensitivity of xerophilic aspergilli and penicillia against alcohol, quaternary ammonium compounds or other possible biocides could help to find the appropriate concentrations and reaction time for the treatments. 

An air sampling was carried out to find out if the fungal contamination inside of the organs leads to a raise of fungal spores in the indoor air and if a possible health risk exists for visitors or for the organist. Also here, SA was used to detect slow growing halotolerant species. The fungi that dominated inside of the organs were only detected in air measurements in close vicinity to the organ and only when the organ was played. It can be concluded that the fungal contamination of the organs do not pose a health risk to the visitors of the church. However, the organists might be subjected to raised levels of fungal mycelial fragments when actively playing the organ. A cleaning of the organ is thus not only recommended for the preservation of the instruments but also for health reasons.

From the results of this study the following major conclusions can be drawn:

- Pipe organs offer a habitat for fungi adapted to low a_w_ values and to low temperatures typical for church climates. Xerophilic aspergilli and penicillia are the most important contaminants in Austrian organs; the fungi have been shown to be halotolerant but not halophilic.
- It is important that the sampling in organs must be carried out using media suitable for the isolation of fungi adapted to low a_w_ values. On DG18 and MEA agar, the slow halotolerant species are overgrown by fast growing air borne fungi and will not be detected. This may lead to a misinterpretation of the microbial situation inside of the organs.
- The climate in pipe organs has to be monitored and adjusted with care and very precisely, because xerotolerant aspergilli and penicillia might thrive under conditions of low water availability that are still not suitable for common air-borne and non-xerophilic fungi.
- Based on a reliable identification (morphological and/or molecular) and a sound knowledge about the ecology of taxonomic groups or single species, fungi are good bioindicators for climate conditions in indoor environments and climatic micro-niches.
- Cleaning of the organs is necessary in order to preserve the instruments, but also because the organist while playing the instrument might be affected by mycelial fragments and spores released into the air.
- A full genome sequencing of the new isolated strains have been performed and will be further analyzed and compared to extremely xerotolerant species as *Eurotium halophiliccum*.

## Figures and Tables

**Table 1:** Fungal isolates from seven different organs in Austrian churches characterized morphologically.

Pipe Organ	Isolated from	Fungi on MEA and DG 18	Fungi on SA (10% NaCl)
**Bad Ischl**	Wood pipes with bolus paint	*Alternaria* sp.	*Aspergillus* sp.
	Case interior	*Aspergillus* sp. *Cladosporium* sp.	*Penicillium* sp.
**Eggelsberg**	Wood pipes	*Penicillium chrysogenum*	*Aspergillus* sp.
	Case interior	*Fusarium* sp.	*Aspergillus* sp. *Penicillium rubens*
	Tracks		*Aspergillus* sp. *Penicillium rubens*
**Nussbach**	Case interior	*Acremonium* sp. *Aspergillus* sp. *Chaetomium* sp.	*Aspergillus* sp.
**Schwanenstadt**	Wood pipes with Bolus paint	*Aspergillus* sp. *Cladosporium* sp. *Fusarium* sp.	*Aspergillus* sp.
	Wood pipes	*Penicillium* sp.	*Aspergillus* sp.
**St. Laurenz**	Casing interior	*Penicillium* sp.	*Penicillium* sp.
	Leather joints	*Aspergillus* sp. *Penicillium* sp.	*Aspergillus* sp.
**Heiligenstadt**	Wood pipes with bolus paint	*Alternaria* sp. *Penicillum commune* *Penicillium brevicompactum* *Penicillium expansum*	*Penicillium* sp.
**Waldzell**	Casing outside	*Alternaria* sp. *Penicillium* sp.	*Aspergillus glabripes*/*vitricola*
	Casing interior	*Alternaria* sp. *Penicillium* sp.	*Aspergillus* sp.

**Table 2:** Identification of halotolerant species based on partial 18S-ITSI–5.8S-ITSII sequencing data derived from Sanger sequencing data.

Pipe Organ	Stain No. ACBR Collection	Most Probable Taxon	Similarity	GenBank Accession No.
**Bad Ischl**	MA 6035	*Aspergillus vitricola*	100%	MH424902
**Eggelsberg**	MA 6038	*Aspergillus vitricola*	99%	MH424903
**Schwanenstadt**	MA 6041	*Aspergillus vitricola*	100%	MH424909
	MA 6037	*Aspergillus versicolor*	100%	MH424917
**St. Laurenz**	MA 6036	*Aspergillus vitricola*	98%	MH424908
**Heiligenstadt**	MA 6040	*Penicillium rubens*	100%	MH427724
**Waldzell**	MA 6039	*Aspergillus glabripes*/*A. vitricola*	97%	MH424912
	MA 6042	*Aspergillus vitricola*	99%	MH424911

**Table 3:** Taxonomy assignment based on beta-tubuline, tef1, 18S rDNA and ITS sequences from whole genome data. The closest organisms are shown with similarity values in brackets.

Strain No./Isolated from	Tef1	β-Tubuline	18S NCBI	18S Silva	ITS
MA 6036/St. Laurenz wood	*P. expansum* (97.8%) *P. digitatum* (97.6%) *P. rubens* (96.1%)	*P. arizonense* (95.2%) *P. digitatum* (95%) *P. rubens* (95%)	*P. solitum*	*Penicillium*/*P. solitum* 0.994	*Penicilium* spp. 100%
MA 6037/Schwanenstadt, Bolus paint	*A. nidulans* (94.8%) *A. niger* (95%) *A. niger* (95%)	*A. nidulans* (98.5%) *A. niger* (98.5%) *A. terreus* (98.3)	*A. versicolor* NRRL 238/*A. oryzae* RIB40	*Aspergillus*/*A. oryzae* RIB40 0.989	*A. creber*/*A. versicolor*/*A. nidulans* 100%
MA 6038/Eggelsberg wood	*A. glaucus* (95.4%) *P. digitatum* (92.4%) *P. rubens* (92.6)	*A. niger* (92.7%) *R. emersonii* (92.2%) *A. oryzae* (92.2%)	*A. oryzae* (99.332%)	*Aspergillus*/*A. lentulus* 0.993	*A. glabripes* 99%
MA 6039/Waldzell wood	*A. glaucus* (95.2) *A. niger* (92.6) *A. niger* (92.6)	*R. emersonii* (95.9%) *A. niger* (95.7%) *A. oryzae* (92.2%)	*A. oryzae* (99.332%)	*Aspergillus*/*A. lentulus* 0.993	*A. glabripes* 99%
MA 6040/Heiligenstadt Bolus paint	*P. rubens* (100%) *P. expansum* (97%) *P. digitatum* (97%)	*P. rubens* (100%) *P. expansum* (99.3%) *P. digitatum* (98.6%)	*P. solitum* (99.944%)	*Penicillium*/*P. solitum* 0.999	*P.* spp. 100% (*P. chrysogenum*/*rubens*)
MA 6041/Schwanenstadt wood	*A. nidulans* *A. niger*	*A. versicolor*	*A. versicolor*	*Aspergillus*/*A. versicolor* 1	*A. vitricola* 99%

**Table 4:** Cardinal growth values of four selected strains from pipe organs.

	MA 6036 *Penicillium* sp.	MA 6037 *Aspergillus* sp.	MA 6039 *Aspergillus* sp. (*glabripes*)	MA 6040 *Penicillium rubens*
Temperature opt.	RT	28 °C	15 °C	15–20 °C
NaCL opt.	0%	0%	0%	0%
NaCl max.	20%	25%	15%	25%
Colony diameter under conditions MEA2% after 6 weeks	23 mm	23 mm	20 mm	42 mm

**Table 5:** Viable cell counts of airborne fungal spores in the churches (CFU = colony forming units).

Sampling Site	CFU/m^3^ MEA in Nave		CFU/m^3^ DG 18 in Nave		CFU/m^3^ SA in Nave		CFU/m^3^ MEA Organist Place		CFU/m^3^ DG 18 Organist Place		CFU/m^3^ SA Organist Place		CFU/m^3^ MEA Reference	CFU/m^3^ DG18 Reference
	min	max	min	max	min	max	min	max	min	max	min	max		
Bad Ischl	260	350	410	540	0	0	10	400	10	870	0	120	n.a. due to heavy rainfall	
Eggelsberg	540	650	550	680	0	0	520	640	500	550	0	0	980	960
Nussbach	130	650	50	130	0	0	10	300	0	100	0	0	980	570
Schwanenstadt	150	420	430	610	0	0	390	490	430	620	10	80	450	360
St. Laurenz	670	1160	460	1040	0	0	900	1200	390	620	0	0	800	100
Waldzell	660	800	560	900	0	0	490	580	540	540	0	0	910	1260
Heiligenstadt														
